# Inflammatory remodeling of the ovarian microenvironment in premature ovarian insufficiency: mechanisms and therapeutic opportunities

**DOI:** 10.3389/fimmu.2026.1909202

**Published:** 2026-08-20

**Authors:** Jiao Fu, Qiaoling Wang, Xinyu Long, Xiayu Qian, Yunfeng Jin

**Affiliations:** 1 Department of Obstetrics and Gynecology, Affiliated Hospital of Nantong University, Nantong, China; 2 School of Medicine, Nantong University, Nantong, Jiangsu, China; 3 Nanjing Medical University, Nanjing, China; 4 Maternal and Child Health Care and Family Planning Service Center, Nantong, China

**Keywords:** ferroptosis, NLRP3 inflammasome, ovarian microenvironment, premature ovarian insufficiency (POI), pyroptosis

## Abstract

Premature ovarian insufficiency (POI) is increasingly recognized as a disorder shaped not only by follicle-intrinsic defects but also by disruption of the ovarian tissue microenvironment. This Mini Review reframes POI as a process of inflammatory ovarian remodeling, in which immune activation, macrophage polarization, mitochondrial stress, inflammasome signaling, and inflammatory regulated cell death interact to accelerate follicular injury and ovarian reserve decline. Rather than providing a pathway-by-pathway summary, we highlight a stepwise model linking upstream genetic, iatrogenic, autoimmune, oxidative, and metabolic insults to immune-cell remodeling, granulosa cell dysfunction, oocyte damage, and follicular loss. Particular attention is given to the macrophage–mitochondria–NLRP3 axis and to the emerging role of pyroptosis and ferroptosis as amplifiers of ovarian inflammation. We also discuss how cell-based therapy, metabolic intervention, mitochondrial protection, inflammasome inhibition, and ferroptosis blockade may restore microenvironmental homeostasis. Finally, we emphasize that current evidence remains largely preclinical and that human samples, spatial omics, and longitudinal studies are needed to validate inflammatory remodeling programs across different POI subtypes.

## 1 Introduction

Premature ovarian insufficiency (POI) is a clinically and biologically heterogeneous disorder characterized by impaired ovarian function before the age of 40 years (1–3). It is commonly manifested by menstrual irregularities or amenorrhea, elevated follicle-stimulating hormone (FSH) levels, reduced estrogen production, and a progressive decline in ovarian reserve (4). Because ovarian follicles are the fundamental units responsible for both endocrine function and female fertility, accelerated follicular depletion or dysfunction represents a central pathological feature of POI (5–8). Traditionally, the pathogenesis of POI has been interpreted mainly from the perspective of follicle-intrinsic injury, including genetic abnormalities, meiotic defects, chemotherapy- or radiation-induced gonadotoxicity, mitochondrial dysfunction, oxidative stress, and apoptosis of granulosa cells or oocytes. However, in idiopathic POI, these upstream insults may be subtle, multifactorial, or clinically unrecognized, making the initiating triggers of ovarian microenvironmental inflammation difficult to define.

However, this follicle-centered view may not fully explain the complexity of POI progression. Ovarian follicles do not develop in isolation; instead, they are embedded within a dynamic tissue microenvironment composed of granulosa cells, theca cells, stromal cells, endothelial cells, immune cells, extracellular matrix components, and soluble mediators (9–12). These cellular and molecular components collectively regulate follicle activation, growth, survival, atresia, steroidogenesis, and tissue repair. Therefore, disruption of the ovarian microenvironment may amplify primary follicular injury and promote a self-perpetuating cycle of inflammation, cellular stress, and follicular loss.

Recent evidence supports the concept that POI involves not only depletion of follicles but also remodeling of the ovarian microenvironment (13–16). In particular, abnormal communication among immune cells, granulosa cells, oocytes, and stromal cells may contribute to impaired follicular homeostasis. Single-cell RNA sequencing has provided important support for this tissue-level view. Han et al. analyzed follicular microenvironments from women with POI, normal ovarian reserve, and advanced maternal age, revealing altered cellular composition and intercellular communication patterns in POI (4). As human observational evidence, this study suggests that POI is associated with coordinated changes across multiple ovarian cell populations rather than isolated damage to a single cell type. However, because such single-cell studies are generally cross-sectional, they cannot determine whether immune remodeling is an initiating event, an amplifier, or a consequence of follicular injury. These findings are especially important because they shift the understanding of POI from a purely follicular degenerative condition toward a microenvironmental disorder involving immune activation and disrupted cellular crosstalk.

Within this framework, inflammation may serve as a key organizing mechanism linking diverse upstream insults to ovarian functional decline (17, 18). Immune-cell activation, macrophage polarization, inflammasome signaling, mitochondrial stress, oxidative damage, pyroptosis, and ferroptosis may converge to damage granulosa cells and oocytes, impair steroidogenesis, and accelerate follicular atresia (19, 20). Therefore, an ovarian niche-centered perspective may help explain how diverse upstream insults converge on common patterns of immune activation, cellular stress, and progressive follicular loss. This perspective provides a useful conceptual basis for exploring inflammatory pathways in POI and for identifying therapeutic strategies aimed at restoring ovarian microenvironmental homeostasis.

In idiopathic POI, the initiating events that trigger inflammatory remodeling of the ovarian microenvironment remain incompletely understood (21, 22). The absence of an identifiable clinical cause does not necessarily indicate the absence of upstream biological stress. Instead, idiopathic POI may reflect the cumulative effects of subtle or subclinical insults that are difficult to capture at the time of diagnosis. Potential initiating triggers include low-grade autoimmune activation, genetic or epigenetic susceptibility, mitochondrial quality-control defects, oxidative stress, metabolic imbalance, environmental toxicant exposure, and early stromal or vascular dysfunction (1, 23–25). These factors may initially produce mild granulosa cell or oocyte stress, mitochondrial damage, ROS accumulation, mtDNA release, or local DAMP production. Such stress signals can then activate resident immune cells, promote macrophage remodeling, and initiate inflammasome-related inflammatory amplification within the ovarian niche (4, 26). Importantly, these triggers are unlikely to act in isolation. In idiopathic POI, inflammatory remodeling may arise from the convergence of multiple weak insults rather than a single dominant cause. For example, a genetically susceptible ovarian niche may become more vulnerable to oxidative or metabolic stress, while subclinical immune imbalance may lower the threshold for macrophage activation and NLRP3 inflammasome signaling. This model may help explain why idiopathic POI is clinically heterogeneous and why the temporal sequence from early ovarian stress to overt ovarian insufficiency remains difficult to define. Future studies using longitudinal sampling, single-cell and spatial multi-omics, immune profiling, and exposure-history assessment are needed to identify the earliest inflammatory events in idiopathic POI.

### 1.1 Evidence levels: human observations and preclinical mechanistic studies

The evidence discussed in this review should be interpreted according to its source and level of validation. Human studies, including single-cell transcriptomic analyses of follicular microenvironments from women with POI, provide important observational evidence that POI is associated with altered cellular composition and disrupted intercellular communication. However, these studies are generally limited in sample size and are often cross-sectional, making it difficult to establish causal or temporal relationships. In contrast, most mechanistic evidence for macrophage activation, mitochondrial dysfunction, NLRP3 inflammasome activation, pyroptosis, ferroptosis, and therapeutic intervention is derived from animal models or cellular systems. These preclinical studies are valuable for identifying candidate pathways, but their findings should not be directly extrapolated to human POI without further validation. Therefore, throughout this review, we distinguish human observational evidence from animal and cell-based mechanistic evidence where appropriate.

## 2 Immune cell activation and macrophage remodeling in POI

Following upstream ovarian stress, immune cell activity within the ovarian microenvironment may represent an early amplifier of tissue injury in POI, including idiopathic cases in which the initial trigger is not clinically evident (27, 28). Among immune populations, macrophages play a particularly central role, both as mediators of inflammation and as modulators of tissue repair. Unlike lymphocytes or NK cells, whose contributions to POI remain less clearly defined, macrophages are abundant within the ovarian stroma and directly interact with granulosa cells, theca cells, and oocytes. Their activation state can profoundly influence follicular survival, steroidogenesis, and overall ovarian homeostasis.

### 2.1 Macrophage activation as a driver of ovarian failure

Preclinical evidence suggests that macrophage activation may contribute to ovarian dysfunction, although the strength and applicability of this evidence vary across models. Bravo et al. used a bmp15-deficient zebrafish model, rather than a mouse model, and showed that disruption of the follicular niche was associated with macrophage activation and ovarian failure-like phenotypes (29). This study provides useful cross-species evidence that altered follicular signaling can reshape the local immune microenvironment. However, because zebrafish ovarian biology does not fully recapitulate mammalian ovarian physiology or the heterogeneity of human POI, this model should be interpreted as mechanistic support for immune–follicular crosstalk rather than direct evidence for human POI. Therefore, the BMP15-related findings are best viewed as one preclinical example suggesting that follicular niche disruption may promote macrophage activation and inflammatory amplification. Additional preclinical studies support the broader concept that macrophage-associated inflammation is involved in ovarian injury and repair. For example, several POI or ovarian toxicity models have linked ovarian dysfunction to macrophage inflammation, macrophage polarization, or macrophage-associated granulosa-cell stress (30, 31). These include studies involving PI3K/Akt/NF-κB-related suppression of macrophage inflammation, MIF/CD74-mediated macrophage polarization, macrophage-associated granulosa-cell senescence during chemotherapy, and SIRT5–RAC2-driven monocyte-to-macrophage differentiation (30, 32). Together with PMSC- and metformin-related studies showing modulation of macrophage polarization and inflammatory signaling, these findings suggest that macrophage remodeling is a recurrent feature across several preclinical ovarian injury contexts, although direct validation in human POI remains limited (33, 34). Such mechanistic insights emphasize the importance of macrophage-driven inflammation in the early stages of POI and provide a rationale for therapeutic strategies targeting these immune populations. Nevertheless, several limitations should be noted. Evidence linking macrophage activation to POI is still mainly derived from animal models, and different models may represent distinct aspects of ovarian dysfunction rather than the full clinical spectrum of human POI. In particular, chemotherapy-induced, genetic, autoimmune, and idiopathic POI may not share identical immune programs. Therefore, macrophage activation should be considered an important candidate mechanism rather than a universal driver of all POI subtypes. Nevertheless, most evidence linking macrophage activation to ovarian dysfunction remains preclinical. Human POI is etiologically heterogeneous, and it remains unclear whether the macrophage programs observed in zebrafish, mouse, or rat models are conserved across idiopathic, autoimmune, genetic, and iatrogenic forms of human POI.

### 2.2 Macrophage polarization and ovarian repair

Macrophage function is highly plastic and can be modulated toward pro-inflammatory (M1) or reparative (M2) phenotypes, with substantial consequences for ovarian tissue integrity. In POI models, strategies that promote M2 polarization have shown promise in mitigating ovarian injury (32, 35–38). Several preclinical interventions further suggest that macrophage phenotype is functionally linked to downstream granulosa-cell inflammatory injury. Rather than representing isolated therapeutic observations, PMSC- and metformin-related findings converge on a shared immune-metabolic axis characterized by attenuation of M1-like inflammatory signaling, enhancement of reparative macrophage features, suppression of inflammasome-related activation, and improved granulosa-cell survival. These findings support the concept that macrophage remodeling may act upstream of both immune amplification and granulosa-cell stress in the ovarian niche. These findings suggest that macrophage phenotype may influence ovarian microenvironmental outcomes: an M1-dominated milieu promotes sustained inflammation and follicular loss, whereas M2 polarization fosters immune regulation, tissue repair, and restoration of ovarian function.

**Figure 1:**
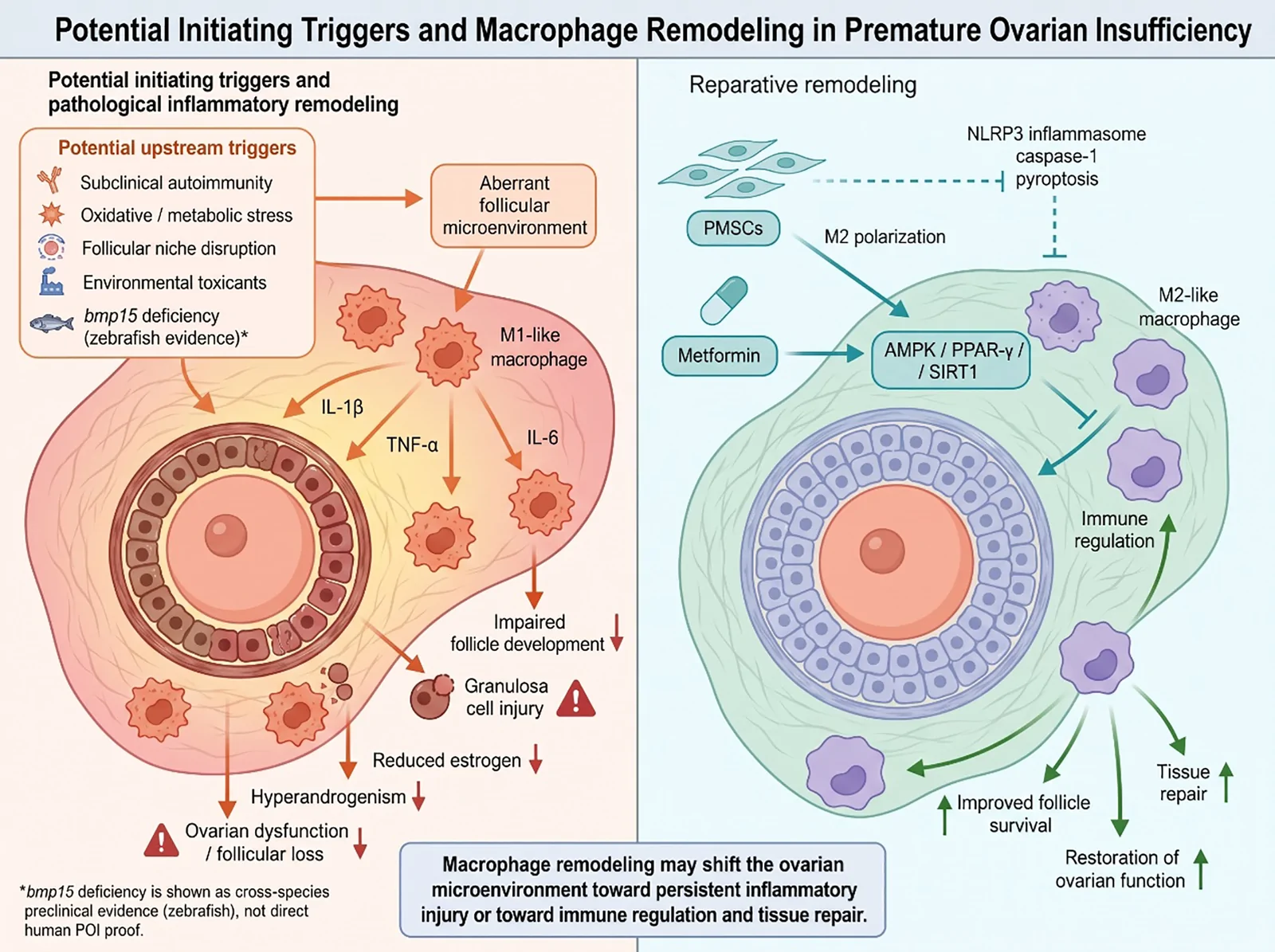
Macrophage activation and remodeling in the ovarian microenvironment during premature ovarian insufficiency. Multiple preclinical and potential upstream triggers, including follicular niche disruption, chemotherapy-induced ovarian injury, oxidative or metabolic stress, autoimmune/inflammatory cues, and bmp15 deficiency in zebrafish, may promote immune-cell activation and macrophage remodeling within the ovarian microenvironment. Activated M1-like macrophages can amplify inflammatory cytokine production, DAMP release, granulosa-cell injury, follicular dysfunction, and ovarian reserve decline. Conversely, PMSC- and metformin-related interventions may promote reparative remodeling by enhancing M2-like polarization, suppressing inflammasome/pyroptotic signaling, and protecting granulosa cells. The bmp15-deficient zebrafish model is presented as cross-species preclinical evidence and should not be interpreted as direct proof of a BMP15-centered mechanism in human POI.

Taken together, these studies suggest that the balance of macrophage activation and polarization may represent an important candidate regulator of ovarian inflammatory remodeling in POI. Therapeutic approaches that selectively modulate macrophage phenotypes hold potential for preventing or reversing ovarian microenvironmental remodeling, highlighting macrophages not only as biomarkers of ovarian health but also as targets for intervention. **Figure 1** summarizes the dual role of macrophages in POI. Activated M1-like macrophages amplify inflammatory injury within the ovarian microenvironment, whereas therapeutic reprogramming toward a reparative phenotype may help restore follicular homeostasis and ovarian function. Importantly, macrophage remodeling should not be viewed as an isolated immune event. Macrophage-derived cytokines, DAMPs, and oxidative mediators may impose additional stress on granulosa cells and oocytes, thereby linking immune activation to mitochondrial dysfunction and inflammasome signaling. This provides the mechanistic basis for the NLRP3–pyroptosis axis discussed in the next section.

## 3 NLRP3 inflammasome and pyroptosis in granulosa cell injury

Following immune-cell remodeling, granulosa cells represent a major effector compartment through which inflammatory stress is translated into follicular dysfunction (4, 39). These cells provide metabolic, endocrine, and paracrine support for oocyte growth and follicle maturation; therefore, inflammatory injury to granulosa cells can disrupt steroidogenesis, impair oocyte competence, and accelerate follicular atresia. In this context, the NLRP3 inflammasome acts as a molecular bridge between upstream immune-metabolic stress and downstream inflammatory cell death (40, 41). Activation of the NLRP3–caspase-1–GSDMD axis promotes IL-1β/IL-18 release and pyroptosis, thereby converting localized granulosa cell stress into a self-amplifying inflammatory loop within the ovarian microenvironment (39, 42, 43).

### 3.1 NLRP3 inflammasome links inflammatory stress to follicular dysfunction

In POI models, NLRP3 inflammasome activation may provide a mechanistic bridge between inflammatory stress and follicular dysfunction (43, 44). Once activated in granulosa cells or immune cells within the ovary, the NLRP3–caspase-1–GSDMD axis can directly promote granulosa cell pyroptosis. Loss of granulosa cell support compromises oocyte survival, disrupts estrogen synthesis, and accelerates follicular degeneration. At the same time, IL-1β and IL-18 released during inflammasome activation may further recruit and activate immune cells, thereby reinforcing a self-amplifying inflammatory loop within the ovarian microenvironment.

Several preclinical POI studies support this mechanistic convergence. Interventions that modulate immune-cell phenotype or restore metabolic homeostasis frequently reduce NLRP3/caspase-1/GSDMD-related signaling and improve ovarian reserve-related phenotypes (33, 40). These findings are better interpreted as evidence for a shared macrophage–mitochondria–inflammasome axis rather than as independent, unrelated observations. In this model, macrophage-derived inflammatory mediators, mitochondrial ROS, metabolic stress, and DAMP release may lower the threshold for NLRP3 activation, while restoration of immune or metabolic balance may restrain inflammasome-driven pyroptotic amplification (40, 45). Evidence from broader inflammasome biology also supports this link between mitochondrial stress, inflammasome activation, gasdermin-mediated pyroptosis, and inflammatory mediator release.

These studies collectively suggest that NLRP3-mediated pyroptosis may be more than a downstream marker of ovarian injury and could participate in inflammatory amplification during POI progression. In this model, chemotherapy, oxidative stress, metabolic imbalance, or immune activation may trigger inflammasome assembly; activated inflammasomes then induce granulosa cell pyroptosis and inflammatory cytokine release; and the resulting inflammatory microenvironment further accelerates follicular loss. Therefore, the NLRP3 inflammasome may represent a candidate convergence point through which diverse ovarian insults are linked to microenvironmental inflammatory damage. However, the causal hierarchy of this pathway remains incompletely defined. In many studies, NLRP3 activation, cytokine release, and granulosa cell pyroptosis are observed together after ovarian injury, making it difficult to determine whether inflammasome activation is an initiating event, an amplifying mechanism, or a downstream consequence of cellular damage (33, 39, 41). More precise genetic and pharmacological approaches, ideally combined with time-course analyses, are needed to clarify the temporal relationship among mitochondrial stress, inflammasome activation, pyroptosis, and follicular loss. It should be noted that the NLRP3–pyroptosis axis in POI is supported mainly by animal and cell-based studies. Although these models provide mechanistic insight into granulosa cell injury, they do not fully recapitulate the etiological heterogeneity of human POI. Human validation using ovarian tissue, follicular fluid, immune profiling, and spatial multi-omics remains limited.

### 3.2 Mitochondrial stress as an upstream trigger of inflammasome activation

Mitochondrial dysfunction is one of the most important upstream triggers of NLRP3 inflammasome activation in injured tissues, including the ovary. Under physiological conditions, mitochondria maintain ATP production, redox balance, and metabolic support for follicular development. However, under pathological stress, damaged mitochondria can produce excessive reactive oxygen species, lose mitochondrial membrane potential, release mitochondrial DNA, and disturb cellular energy metabolism (41, 46–51). These events create a pro-inflammatory intracellular environment that facilitates NLRP3 inflammasome activation.

Yin et al. provided experimental support for the link between mitochondrial damage and NLRP3 inflammatory activation in a cyclophosphamide-induced POI rat model (52). Their study showed that intervention with moxibustion alleviated mitochondrial dysfunction and reduced NLRP3 inflammasome activation, suggesting that mitochondrial protection may help interrupt the inflammatory cascade leading to ovarian insufficiency. Although the therapeutic modality itself requires further validation and mechanistic refinement, the study supports an important biological axis: mitochondrial stress may serve as a proximal signal that activates NLRP3, promotes inflammatory injury, and contributes to ovarian functional decline.

From a mechanistic perspective, the mitochondrial stress–NLRP3 axis provides a useful framework for understanding how non-immune injuries evolve into inflammatory microenvironmental remodeling. Initial insults such as chemotherapy, oxidative stress, or metabolic disturbance may first damage mitochondria in granulosa cells. Mitochondrial dysfunction then promotes ROS accumulation and inflammasome activation, which induces pyroptosis and cytokine release. These inflammatory signals subsequently affect neighboring granulosa cells, oocytes, stromal cells, and macrophages, transforming localized cellular stress into broader ovarian tissue dysfunction. Thus, targeting mitochondrial homeostasis may indirectly suppress inflammasome activation and preserve follicular microenvironmental stability.

**Figure 2:**
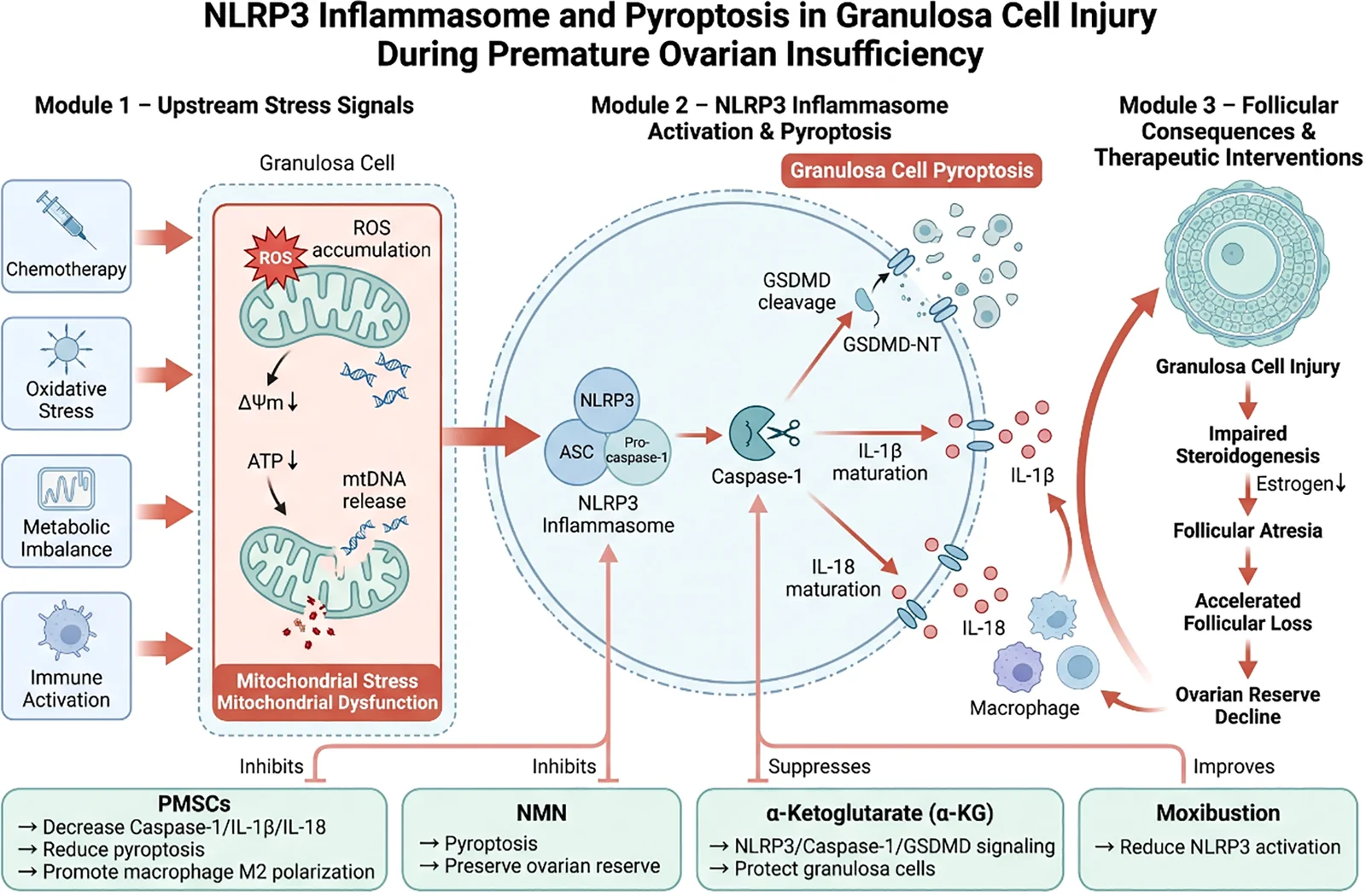
NLRP3 inflammasome activation and pyroptosis in granulosa cell injury during premature ovarian insufficiency. Chemotherapy, oxidative stress, metabolic imbalance, and immune activation induce mitochondrial dysfunction in granulosa cells, which triggers NLRP3 inflammasome activation. This leads to caspase-1 activation, GSDMD-mediated pyroptosis, and IL-1β/IL-18 release, thereby amplifying ovarian inflammation and follicular loss. PMSCs, NMN, α-KG, and mitochondrial-protective interventions may suppress this pathway and help preserve ovarian reserve.

Overall, current evidence indicates that the NLRP3 inflammasome and pyroptosis are central mediators of granulosa cell injury in POI. By connecting mitochondrial stress, immune activation, inflammatory cytokine release, and follicular loss, this pathway provides both a mechanistic explanation for ovarian microenvironmental remodeling and a therapeutic target for preserving ovarian reserve. **Figure 2** illustrates how mitochondrial stress acts as an upstream trigger of NLRP3 inflammasome activation in granulosa cells, leading to pyroptosis and inflammatory amplification in POI. It also highlights potential therapeutic strategies that preserve ovarian reserve by suppressing inflammasome signaling or restoring mitochondrial homeostasis.

## 4 Regulated cell death and inflammatory amplification: from pyroptosis to ferroptosis

While classical apoptosis has long been considered the primary mode of follicular cell death in POI, recent evidence suggests that inflammatory forms of regulated cell death, particularly pyroptosis and ferroptosis, may also contribute to ovarian microenvironmental remodeling. These two pathways differ in their molecular execution: pyroptosis is mainly driven by inflammasome activation and gasdermin-mediated membrane pore formation, whereas ferroptosis is characterized by iron-dependent lipid peroxidation and redox imbalance. However, both pathways can amplify tissue injury by promoting DAMP release, inflammatory signaling, oxidative damage, and follicular cell dysfunction. Therefore, pyroptosis and ferroptosis are discussed here as complementary mechanisms linking cellular stress to inflammatory amplification and follicular loss in POI.

### 4.1 Pyroptosis as an inflammatory form of granulosa cell death

Pyroptosis, driven primarily by the NLRP3–caspase-1–GSDMD axis, represents a form of regulated, lytic cell death that releases potent pro-inflammatory mediators such as IL-1β and IL-18. In granulosa cells, pyroptosis not only leads to cell loss but also exacerbates the local inflammatory milieu, creating a positive feedback loop that further impairs neighboring follicles (53, 54). Experimental studies indicate that interventions targeting this pathway can mitigate ovarian injury. For example, placental mesenchymal stem cells suppress granulosa cell pyroptosis by inhibiting NLRP3 activation, thereby improving follicular survival. Similarly, nicotinamide mononucleotide (NMN) and α-ketoglutarate (α-KG) have been shown to protect ovarian reserve by attenuating NLRP3-mediated pyroptosis in chemotherapy-induced POI models (55). Collectively, these findings highlight that controlling pyroptosis is critical not only for preserving granulosa cell viability but also for restraining inflammatory amplification within the ovarian microenvironment. However, pyroptosis does not fully explain the spectrum of inflammatory cell death in POI. The same upstream stressors that activate inflammasome signaling, such as mitochondrial dysfunction, ROS accumulation, metabolic disturbance, and oxidative damage, may also disrupt iron handling and lipid redox homeostasis. This provides a mechanistic bridge from cytokine-amplifying pyroptosis to ferroptosis, a redox-driven form of cell death that may further aggravate oocyte and follicular injury.

### 4.2 Ferroptosis as a redox-inflammatory mechanism in POI

In this context, ferroptosis represents another inflammatory injury pathway that complements pyroptosis by linking oxidative stress, iron-dependent lipid peroxidation, and redox imbalance to follicular damage (56–60). Although traditionally studied in the context of cancer and neurodegeneration, recent studies have identified its relevance in ovarian biology. Wang et al. demonstrated that deficiency of BNC1 in mice triggers ferroptosis in oocytes via the NF2–YAP pathway, leading to premature ovarian insufficiency (61). Pharmacologic inhibition of YAP or ferroptosis partially rescued ovarian function, underscoring the contribution of redox-mediated cell death to follicular loss. Ferroptotic death may further exacerbate ovarian inflammation by releasing oxidized lipids and damage-associated molecular patterns (DAMPs), thereby integrating oxidative stress with immune activation in the ovarian niche.

Taken together, pyroptosis and ferroptosis represent two interconnected but mechanistically distinct forms of inflammatory regulated cell death in POI. Pyroptosis mainly translates inflammasome activation into cytokine release, membrane rupture, and inflammatory amplification, whereas ferroptosis converts iron-dependent lipid peroxidation and redox imbalance into oocyte and follicular injury. Importantly, these pathways may be connected by shared upstream triggers, including mitochondrial dysfunction, ROS accumulation, metabolic stress, and oxidative damage. Both pathways can promote DAMP release and inflammatory feedback, thereby disrupting follicular homeostasis and accelerating ovarian reserve depletion. This integrated view expands the understanding of POI beyond classical apoptosis and suggests that combined targeting of inflammasome-mediated pyroptosis and redox-driven ferroptosis may provide a more comprehensive strategy for preserving ovarian function. These interconnected mechanisms also clarify why therapeutic strategies for POI should not be limited to endocrine replacement or single-cell protection. Instead, effective intervention may require interruption of the inflammatory remodeling circuit at multiple levels, including immune-cell activation, mitochondrial stress, inflammasome signaling, and ferroptosis-associated lipid peroxidation. The following section therefore discusses therapeutic opportunities according to these mechanistic entry points. Compared with apoptosis and inflammasome-associated pyroptosis, evidence for ferroptosis in POI remains relatively recent and less extensive. Most studies have focused on selected injury models or specific molecular contexts, and it remains unclear whether ferroptosis is broadly involved across different POI etiologies. In addition, ferroptosis markers can overlap with oxidative stress responses, making rigorous validation with multiple biochemical, genetic, and rescue approaches essential.

## 5 Therapeutic strategy categories targeting inflammatory ovarian microenvironment remodeling

Based on the inflammatory remodeling model discussed above, emerging therapeutic approaches for POI can be organized into four mechanistic strategy categories: immune-niche remodeling, metabolic–mitochondrial restoration, inflammasome/pyroptosis inhibition, and ferroptosis/redox-inflammatory modulation. These strategies should not be viewed as isolated interventions (33, 34). Instead, they target different nodes of the same pathological circuit, in which immune activation, mitochondrial dysfunction, inflammatory cell death, and follicular injury reinforce each other (57, 61). This category-based framework helps clarify how cell-based therapies, metabolic regulators, inflammasome-targeted approaches, and ferroptosis-related interventions may contribute to restoration of ovarian microenvironmental homeostasis. Importantly, the therapeutic approaches discussed below are supported predominantly by preclinical evidence. Their clinical relevance to human POI remains uncertain until validated by well-designed human studies.

### 5.1 Immune-niche remodeling by cell-based immunomodulation

Cell-based therapy represents one of the most promising strategies for modulating the inflammatory ovarian microenvironment (27, 28, 62–64). Among these approaches, placental mesenchymal stem cells (PMSCs) have attracted attention because of their immunoregulatory and tissue-reparative properties. Chen et al. demonstrated that PMSCs improved ovarian function in a POI model by suppressing NLRP3 inflammasome activation, reducing caspase-1 activity, and decreasing the production of IL-1β and IL-18 (33). Importantly, PMSCs also promoted macrophage polarization toward an M2-like reparative phenotype, suggesting that their protective effect is not limited to direct support of ovarian cells but also involves reprogramming of the immune microenvironment (33). This finding is important for two reasons. First, it links macrophage phenotype, inflammasome activity, and granulosa cell injury within a unified therapeutic framework. Second, it suggests that successful POI treatment may require restoration of tissue-level immune balance rather than simple suppression of inflammation. By shifting macrophages away from a pro-inflammatory state and toward a repair-associated phenotype, PMSCs may help interrupt the cycle of immune activation, cytokine release, granulosa cell death, and follicular loss. Therefore, PMSCs and related cell-based approaches can be categorized as immune-niche remodeling strategies in POI. Their major therapeutic rationale is to shift the ovarian microenvironment from a pro-inflammatory state toward a reparative state by modulating macrophage polarization, suppressing inflammasome activation, and protecting granulosa cells. However, their translational application remains limited by unresolved questions regarding long-term safety, optimal delivery route, dosing strategy, persistence in ovarian tissue, and reproducibility in human POI.

### 5.2 Metabolic–mitochondrial restoration

Metabolic and mitochondrial interventions are another important therapeutic direction. Ovarian follicles are highly metabolically active structures, and granulosa cells depend on coordinated mitochondrial function, redox balance, NAD+ metabolism, and energy production to support oocyte development and steroidogenesis. In POI, chemotherapy, oxidative stress, and inflammatory activation can disrupt these metabolic networks, leading to mitochondrial dysfunction and activation of inflammatory pathways such as the NLRP3 inflammasome. Therefore, metabolic therapies should not be understood simply as antioxidants; rather, they may reshape the ovarian microenvironment by restoring mitochondrial homeostasis, limiting inflammasome activation, and reducing inflammatory cell death (65–68). Several recent studies support this concept. Nicotinamide mononucleotide (NMN), a precursor of NAD+ metabolism, has been shown to improve ovarian reserve in cyclophosphamide-induced POI mice by inhibiting NLRP3-mediated pyroptosis (40). This suggests that restoration of metabolic resilience may suppress inflammatory cell death and preserve follicular integrity. Similarly, α-ketoglutarate (α-KG), a key tricarboxylic acid cycle intermediate, improved ovarian reserve function in POI rats by inhibiting NLRP3-, caspase-1-, and GSDMD-associated pyroptotic signaling in granulosa cells (69). These findings highlight the possibility that metabolic intermediates may directly influence inflammatory signaling within the ovarian niche (69).

Metformin also provides an important example of metabolic-inflammatory intervention. Yang et al. reported that metformin protected granulosa cells in chemotherapy-induced premature ovarian failure through the AMPK/PPAR-γ/SIRT1 pathway and reduced M1 macrophage-associated inflammatory damage (34). This mechanism is particularly relevant because AMPK and SIRT1 are closely linked to cellular energy sensing, mitochondrial quality control, oxidative stress regulation, and inflammatory restraint (34). Thus, metformin may protect the ovary not only by improving metabolic stress responses but also by dampening immune-mediated microenvironmental injury. Together, NMN, α-KG, and metformin suggest that metabolic regulation and immune modulation are tightly connected in POI therapy.

### 5.3 Targeting inflammatory cell death

Because pyroptosis and ferroptosis contribute to inflammatory amplification and follicular loss, targeting regulated inflammatory cell death may represent a future therapeutic strategy for POI (70–72). The NLRP3–caspase-1–GSDMD pathway is particularly attractive because it connects mitochondrial stress, immune activation, inflammatory cytokine release, and granulosa cell pyroptosis. Pharmacological inhibition of NLRP3, caspase-1, GSDMD pore formation, or IL-1β/IL-18 signaling may theoretically reduce granulosa cell loss and prevent the spread of inflammation within the ovarian microenvironment. Although direct clinical evidence in POI is still lacking, preclinical findings from PMSCs, NMN, and α-KG studies support the concept that suppressing NLRP3-mediated pyroptosis can improve ovarian reserve.

In addition to pyroptosis, ferroptosis may provide another therapeutic entry point. Wang et al. showed that BNC1 deficiency induced oocyte ferroptosis through the NF2–YAP pathway and contributed to POI-like phenotypes (61). Importantly, pharmacological inhibition of YAP or ferroptosis partially rescued ovarian dysfunction, suggesting that ferroptosis inhibition may have follicle-protective potential (61). This mechanism expands the therapeutic landscape from cytokine-centered inflammation to redox-inflammatory injury. By limiting iron-dependent lipid peroxidation and oxidative membrane damage, ferroptosis-targeted interventions may help preserve oocyte viability and follicular structure (57, 58).

Despite these therapeutic possibilities, the translational readiness of microenvironment-targeted interventions remains limited. Most interventions discussed above have been tested in animal models or cellular systems, and few have been validated in well-designed clinical studies of human POI. Important unresolved issues include ovarian specificity, long-term safety, optimal timing, dosing, delivery route, durability of ovarian functional recovery, and potential effects on offspring health. Moreover, because POI is etiologically heterogeneous, therapeutic responses may differ substantially among chemotherapy-induced, autoimmune, genetic, metabolic, and idiopathic POI. Future studies should therefore avoid assuming a single universal therapeutic strategy and instead develop subtype-specific biomarkers and intervention windows.

Overall, therapeutic strategies for POI may need to move from single-target endocrine replacement toward microenvironmental restoration. Cell-based immunomodulation, metabolic and mitochondrial protection, and inhibition of inflammatory cell death represent three interconnected directions. Future studies should determine whether these approaches can be combined, whether they are effective across different causes of POI, and which biomarkers can identify patients most likely to benefit from microenvironment-targeted therapies.

## 6 Conclusions and future perspectives

POI should not be viewed solely as a consequence of isolated follicular depletion or intrinsic damage to oocytes and granulosa cells. Instead, accumulating evidence suggests that POI is closely associated with disruption of the ovarian tissue microenvironment. Within this framework, immune activation, macrophage remodeling, mitochondrial dysfunction, inflammasome activation, pyroptosis, and ferroptosis may interact to create a self-amplifying cycle of inflammation and follicular loss. This perspective helps explain why diverse etiological factors, including genetic defects, chemotherapy, oxidative stress, and metabolic disturbance, can converge on similar outcomes of impaired follicular survival and ovarian reserve decline.

Recent experimental studies have begun to identify key components of this inflammatory remodeling process. Macrophage activation may directly contribute to ovarian dysfunction, whereas macrophage polarization toward reparative phenotypes may support tissue recovery. The NLRP3 inflammasome and downstream pyroptotic signaling provide a mechanistic link between mitochondrial stress, inflammatory cytokine release, granulosa cell injury, and follicular atresia. In parallel, ferroptosis connects lipid peroxidation and redox imbalance to oocyte damage and ovarian insufficiency. Together, these mechanisms suggest that regulated inflammatory cell death may not be merely a consequence of ovarian injury, but could participate in inflammatory amplification and follicular loss during POI progression.

Despite these advances, several limitations should be emphasized. First, human evidence remains relatively limited and is mainly observational, including transcriptomic or single-cell analyses of ovarian or follicular microenvironmental samples. Such studies are valuable for identifying disease-associated cellular and communication changes, but they cannot establish causality or temporal sequence. Second, most mechanistic evidence supporting macrophage activation, mitochondrial dysfunction, NLRP3 inflammasome activation, pyroptosis, ferroptosis, and therapeutic intervention is derived from animal models or cellular systems. These models are useful for pathway dissection, but they may not fully represent the heterogeneity of human POI, including idiopathic, autoimmune, genetic, metabolic, and iatrogenic subtypes. Third, chemotherapy-induced POI models are widely used but may overrepresent acute ovarian injury and may not capture the chronic, multifactorial remodeling process in idiopathic POI. Finally, clinical translation of microenvironment-targeted therapies remains limited by insufficient human validation, lack of long-term safety data, uncertain dosing and delivery strategies, and absence of subtype-specific biomarkers. Future studies integrating human samples, longitudinal clinical cohorts, spatial multi-omics, and mechanistic validation in clinically relevant models are needed to bridge the gap between preclinical findings and human POI.

From a therapeutic perspective, future research should determine whether different causes of POI share common inflammatory microenvironmental programs or require etiology-specific interventions. Strategies such as NLRP3 inhibition, macrophage reprogramming, mesenchymal stem cell-based therapy, metabolic intervention, mitochondrial protection, and ferroptosis modulation are promising in preclinical settings but require rigorous validation in human POI. Importantly, the goal of future treatment should move beyond endocrine replacement alone toward preservation and restoration of ovarian microenvironmental homeostasis. A deeper understanding of inflammatory remodeling in POI may help identify mechanism-based therapeutic opportunities, but these approaches require rigorous validation in human samples and clinically relevant models before they can be translated into patient care.
